# Association between lower limb muscle strength and musculoskeletal ambulation disability symptom complex in patients with medial meniscus posterior root tears

**DOI:** 10.1007/s00590-024-04158-4

**Published:** 2024-12-12

**Authors:** Mikao Fukuba, Koki Kawada, Yuki Okazaki, Yoshimi Katayama, Masanori Hamada, Toshifumi Ozaki, Takayuki Furumatsu

**Affiliations:** 1 Department of Physical Medicine and Rehabilitation, Okayama University Hospital, Okayama, Japan; 2 Department of Orthopaedic Surgery, Okayama University Graduate School of Medicine, Dentistry, and Pharmaceutical Sciences, Okayama, Japan; 3 Department of Orthopaedic Surgery, Japanese Red Cross Okayama Hospital, 2-1-1 Aoe, Kitaku, Okayama, 700-8607 Japan

**Keywords:** Musculoskeletal ambulation disability symptom complex, Meniscus, Posterior root tear, Physical therapy, Rehabilitation

## Abstract

**Purpose:**

In this study, we aimed to evaluate the changes in and the relationship between lower limb muscle strength and physical function before and after medial meniscus posterior root (MMPR) repair.

**Methods:**

Thirty-three patients who underwent MMPR repair were evaluated. Pain was evaluated with the numerical rating scale (NRS), and knee flexor/extensor muscle strength was assessed using a handheld dynamometer. Physical function was evaluated using a timed up and go (TUG) test. The NRS, knee flexor/extensor muscle strength, and TUG were compared preoperatively and 1 year postoperatively using the Wilcoxon signed-rank test. The correlation of patient characteristics, NRS score, knee flexor/extensor muscle strength, and preoperative TUG with the postoperative TUG was analyzed using Spearman’s correlation coefficient.

**Results:**

NRS (3.5 ± 2.1 to 0.1 ± 0.5 points), knee flexor strength (111.9 ± 50.2 to 146.7 ± 51.5 Nm), knee extensor strength (181.9 ± 92.8 to 256.9 ± 107.1 Nm), and TUG (12.3 ± 5.7 to 9.2 ± 2.2 s) all improved significantly from preoperatively to 1 year postoperatively (*p* < 0.001). The postoperative TUG was negatively correlated with the preoperative TUG (r = 0.578, *p* < 0.001), preoperative knee flexor muscle strength (r = − 0.355, *p* = 0.042), preoperative knee extensor muscle strength (r = − 0.437, *p* = 0.010), and postoperative knee extensor muscle strength (r = − 0.478, *p* = 0.004).

**Conclusion:**

In patients undergoing MMPR repair, surgery and rehabilitation significantly improve lower limb muscle strength and physical function. There was a significant correlation between lower limb muscle strength and TUG, and further strengthening of the lower limb muscles from the preoperative level is desirable to improve patients’ physical function further.

**Level of evidence:**

IV.

## Introduction

Medial meniscus posterior root (MMPR) tears (MMPRTs) commonly affect middle-aged individuals, particularly those who experience painful popping sensations in the posteromedial area of the knee during light activities like walking or using stairs [1, 2]. Risk factors for MMPRTs include advanced age, female sex, sedentary lifestyle, overweight, and varus knee alignment [3]. MMPRTs result in a compromised hoop function of the meniscus, leading to increased pressure on the medial compartment and subsequent cartilage deterioration [4]. Meniscal repair has emerged as the preferred treatment for MMPRTs in recent years, with favorable clinical results [5].

Musculoskeletal ambulation disability symptom complex (MADS), a disease concept proposed by the Japanese Orthopaedic Association in 2006, was designed to screen for patients at risk of losing their ability to walk [6]. MADS is diagnosed based on 11 underlying diseases or medical histories, including knee osteoarthritis, as well as physical examination findings that assess lower limb muscle strength and walking ability. The criteria for evaluating physical function in MADS include a “time standing on one leg” of less than 15 s or a “timed up and go (TUG) test” of 11 s or more [7].

MADS is a disease of older individuals that is associated with a decreased ability to walk and move, resulting in increased susceptibility to falls and disabilities in daily living [7]. Appropriate rehabilitation, surgery, and various interventions are required for patients with MADS. In patients with osteoarthritis, both lower limb muscle strength and physical function are impaired before arthroplasty, but effective physical therapy has been reported to improve postoperative physical function [8]. In patients with symptomatic meniscal injuries, a relationship between lower limb muscle strength and physical function has also been reported [9]. In patients with MMPRTs, an association between postoperative quadriceps muscle strength and clinical scores has been shown [10]. However, few studies have been conducted on MADS and the relationship between lower limb muscle strength and physical function in patients with MMPRTs.

This study aimed to evaluate knee flexor and extensor muscle strength and physical function before and after MMPR repair and to determine the relationship between them. We hypothesized that the higher the preoperative or postoperative lower limb muscle strength, the better the postoperative physical function.

## Materials and methods

### Patients

This study was performed following the Declaration of Helsinki and approved by our institution. Written informed consent was obtained from all patients. The criteria for MMPR repair in our practice include a femorotibial angle below 180°, Kellgren–Lawrence grades 0–2, and mild cartilage abnormalities.

**Fig. 1:**
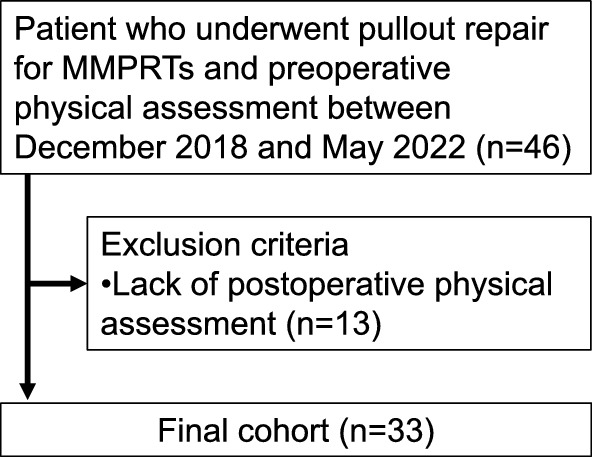
Flowchart of the study protocol. Abbreviations: MMPRT, medial meniscus posterior root tear

Forty-six patients who underwent pullout repair for MMPRTs and preoperative physical assessment between December 2018 and May 2022 were included in this study (Fig. 1). Among them, 13 patients whose physical function could not be measured 1 year postoperatively were excluded, and the remaining 33 patients were evaluated. Patients underwent rehabilitation with a physical therapist for at least 3 months postoperatively, focusing on quadriceps muscle strengthening.

### Rehabilitation protocol

After surgery, knee joint range of motion was initially restricted in extension using a brace for 1 week, after which knee flexion was gradually increased by 30° each week, up to 120° 3 months post-surgery. Weight-bearing was suspended for the first week and then progressively increased by 20 kg each week, allowing most patients to achieve full weight-bearing by 4 weeks.

Physiotherapy began before surgery and continued for at least 3 months postoperatively, with weekly sessions. Emphasis was placed on quadriceps strengthening and included quadriceps setting, straight leg raising training, and seated knee extension exercises, which patients were encouraged to perform independently. After 3 months, half-squat training was introduced. No machine-based exercises were included.

### Muscle strength assessment

**Fig. 2:**
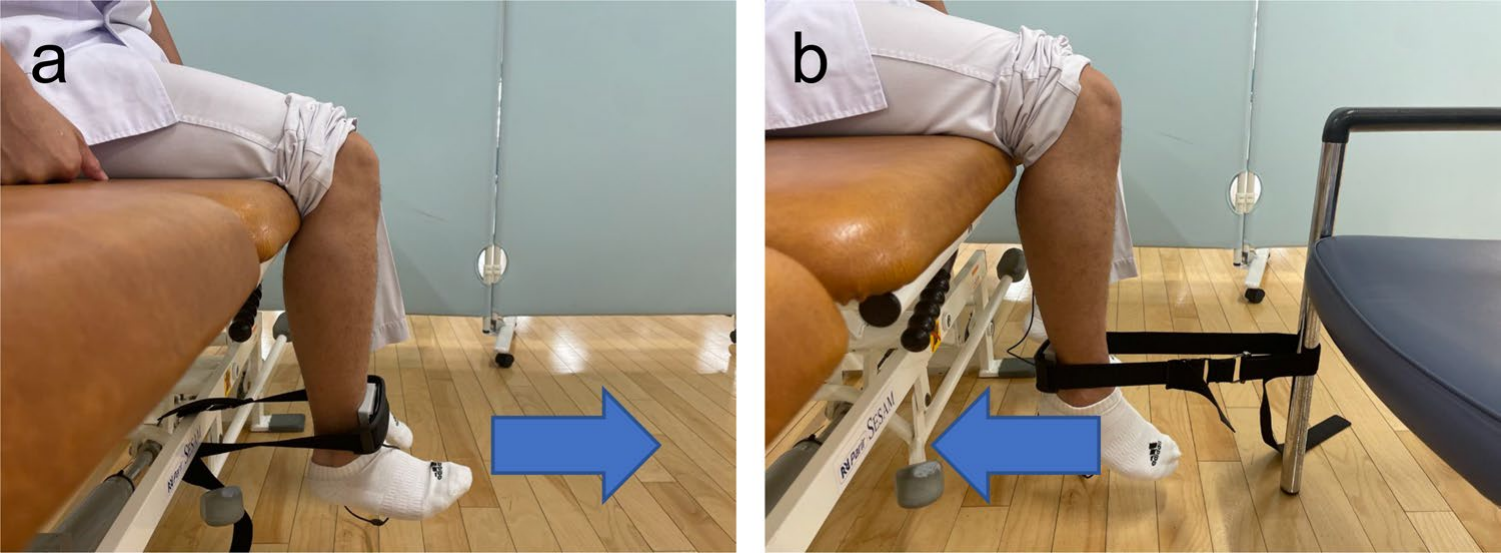
Measurement of knee flexor and extensor muscle strength. **a**: Measurement of the knee extensor muscle strength. The test was performed with the patient in a 90-degree hip/knee joint posture, with one attachment of the instrument placed on the front of the lower leg and the other attached to the side of the bed. **b**: Measurement of the knee flexor muscle strength. The hip and knee joints were measured in the same posture, with one attachment of the instrument placed on the posterior aspect of the lower leg and the other fixed to a chair

The strength of the knee flexor and extensor muscles was measured preoperatively and 1 year postoperatively using a handheld dynamometer (μTas F-1; ANIMA, Tokyo, Japan). The knee extensor muscle strength was measured by placing the instrument on the anterior aspect of the lower leg and extending the knee joint from a 90-degree flexed position (Fig. 2a). Similarly, the knee flexor muscle strength was measured by placing the instrument on the posterior aspect of the lower leg and flexing the knee joint from a 90-degree flexed position (Fig. 2b). Measurements were performed with isometric contraction and ranged from 0 to 980 Nm.

### Clinical assessment

Pain was assessed preoperatively and 1 year postoperatively using the numerical rating scale (NRS), which rates pain on an 11-point scale with values ranging from 0 to 10. On this scale, 0 represents the absence of pain, and 10 indicates the most severe pain imaginable.

### Physical function assessment

**Fig. 3:**
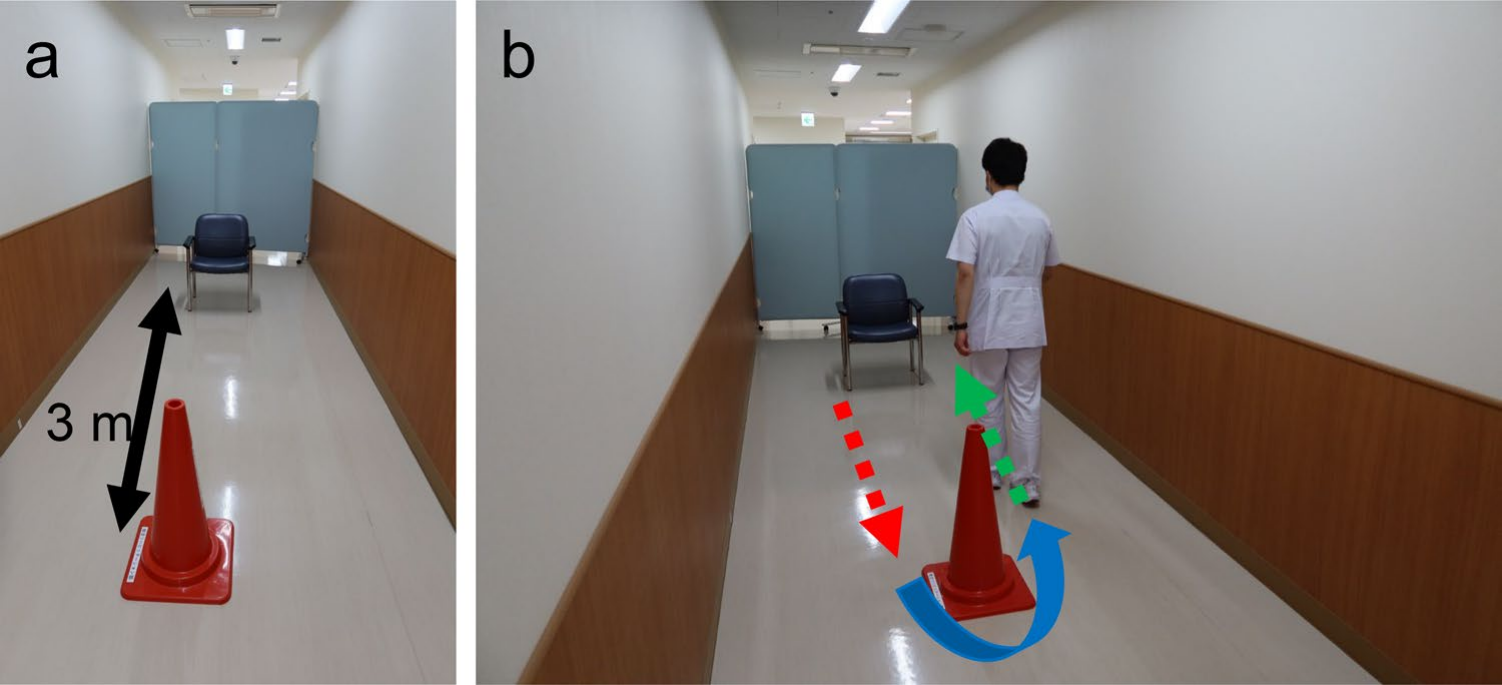
Timed up and go test. **a**: A chair with armrests and a safety cone were set up at 3 m distances. **b**: Patients were first seated in the chair. The total time it took for the patient to get up from the chair (red arrow), move around the safety cone at a comfortable speed (blue curved arrow), and sit down in the chair (green arrow) was measured

The TUG test was used to assess physical function preoperatively and 1 year postoperatively. The TUG test measures the time required to get up from a chair, go around a safety cone, and walk to a chair at 3 m distances (Fig. 3).

### Radiographic assessment

Standing knee joint radiographs and Rosenberg views were taken preoperatively and 1 year postoperatively. The femorotibial angle was evaluated using the standing knee joint radiographs, and the Kellgren–Lawrence grade was evaluated with the Rosenberg view.

### Patients’ activity assessment

The Tegner activity score was evaluated preoperatively and 1 year postoperatively [11].

### Statistical analyses

Statistical analysis was performed using EZR software (Saitama Medical Center, Saitama, Japan). All assessment parameters were evaluated for normal distribution using the Shapiro–Wilk test. Patient characteristics were normally distributed, while the other parameters were not normally distributed. The NRS, knee flexor/extensor muscle strength, and TUG were compared preoperatively and 1 year postoperatively using the Wilcoxon signed-rank test. The correlation of patient characteristics, NRS score, knee flexor/extensor muscle strength, and preoperative TUG with the postoperative TUG was analyzed using Spearman’s correlation coefficient. Statistical significance was defined as *p* < 0.05.

Actual power analysis was performed using the post-hoc test (G*Power, version 3.1.9.6; University of Düsseldorf, Düsseldorf, Germany) to detect power and evaluate the correlation between postoperative knee extensor muscle strength and TUG. In the post-hoc analysis, the actual power was calculated to be 99.9% using an effect size of 0.691, an α-error of 0.05, and a sample size of 33.

## Results

A total of 33 patients were analyzed (15 men and 18 women). The mean age was 64.8 ± 8.5 years, and the mean body mass index was 27.1 ± 3.2 kg/m^2^ (Table 1). There were no patients with low nutritional status, such as hypoalbuminemia.

**Table 1:** Patient characteristics

Characteristic	Value
Patient, n	33
Sex, male/female	15/18
Age (years)	64.8 ± 8.5
Height (m)	1.62 ± 7.13
Body weight (kg)	67.7 ± 9.1
Body mass index (kg/m^2^)	27.1 ± 3.2
Preoperative Kellgren–Lawrence grade, 0/1/2/3/4	0/24/9/0/0
Postoperative Kellgren–Lawrence grade, 0/1/2/3/4	0/16/16/1/0
Preoperative femorotibial angle (°)	178.8 ± 1.4
Postoperative femorotibial angle (°)	179.8 ± 1.6
Preoperative Tegner activity score (points) [range]	2.1 ± 1.0 [0–4]
Postoperative Tegner activity score (points) [range]	3.1 ± 0.7 [2–5]

Values are presented as the mean ± standard deviation or numbers

The knee flexor muscle strength at 1 year postoperatively (146.7 ± 51.5 Nm) was significantly higher than the preoperative value (111.9 ± 50.2 Nm, p < 0.001). Similarly, the knee extensor muscle strength at 1 year postoperatively (256.9 ± 107.1 Nm) was significantly higher than the preoperative value (181.9 ± 92.8 Nm, *p* < 0.001).

The NRS score at 1 year postoperatively (0.1 ± 0.5 points) was significantly improved from the preoperative score (3.5 ± 2.1 points, *p* < 0.001). TUG time at 1 year postoperatively (9.2 ± 2.2 s) was also significantly improved from the preoperative time (12.3 ± 5.7 s, *p* < 0.001).

**Fig. 4:**
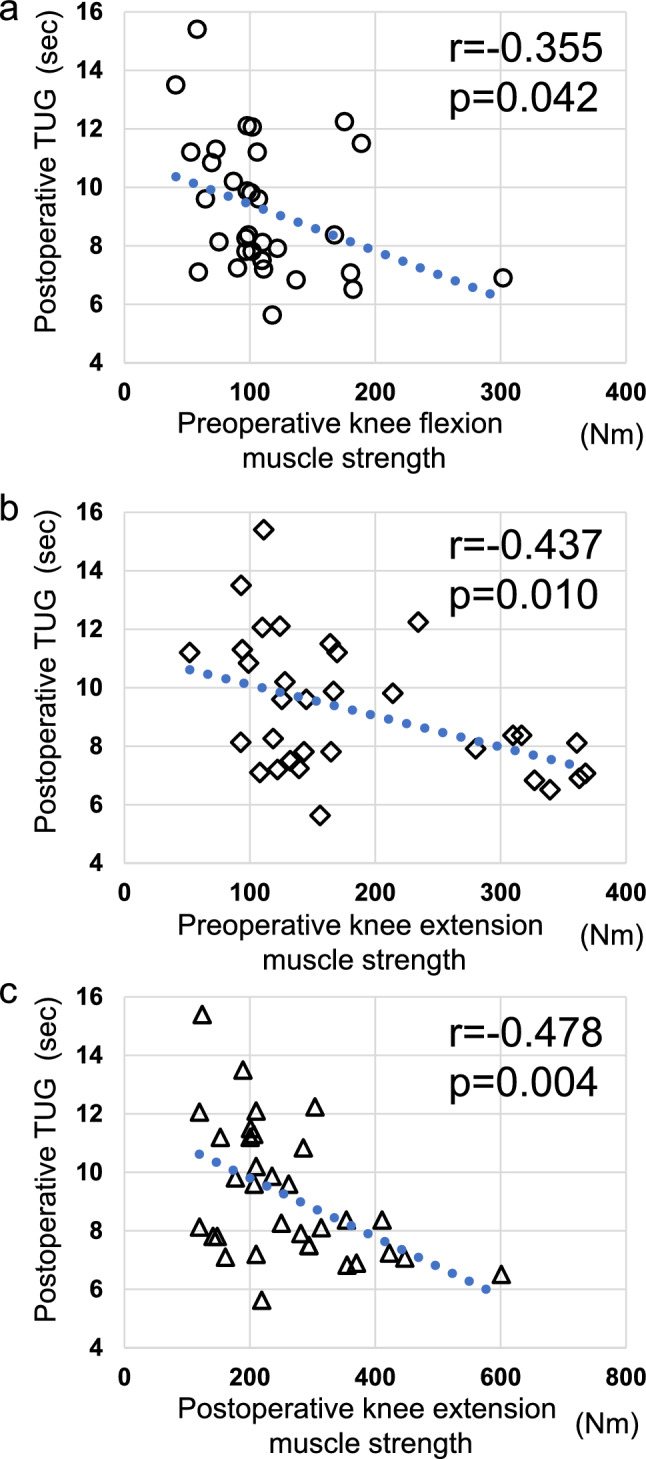
Scatterplot of correlations. **a**: Preoperative knee flexor muscle strength and postoperative TUG show a negative correlation (r = − 0.355, *p* = 0.042). **b**: Preoperative and postoperative knee extensor muscle strength demonstrate a negative correlation (r = − 0.437, *p* = 0.010). **c**: Postoperative knee extensor muscle strength and postoperative TUG show a negative correlation (r = − 0.478, *p* = 0.004). Abbreviation: TUG, timed up and go

Postoperative TUG time was negatively correlated with the preoperative knee flexor muscle strength (correlation coefficient = − 0.355, *p* = 0.042), preoperative knee extensor muscle strength (correlation coefficient = − 0.437, *p* = 0.010), and postoperative knee extensor muscle strength (correlation coefficient = − 0.478, *p* = 0.004; Table 2, Fig. 4). The postoperative TUG time was positively correlated with the preoperative TUG time (correlation coefficient = 0.578, *p* < 0.001).

**Table 2:** Spearman correlation analysis of postoperative TUG

Variable	Postoperative TUG	
	Correlation coefficient	*p* value
Age	0.333	0.058
Height	− 0.286	0.107
Body weight	− 0.305	0.084
Body mass index	− 0.089	0.621
Preoperative NRS	− 0.014	0.939
Preoperative knee flexor muscle	− 0.355	0.042*
Preoperative knee extensor muscle	− 0.437	0.010*
Preoperative TUG	0.578	< 0.001*
Postoperative NRS	0.361	0.059
Postoperative knee flexor muscle	− 0.132	0.503
Postoperative knee extensor muscle	− 0.478	0.004*

NRS, numerical rating scale; TUG, timed up and go

*Statistically significant

## Discussion

The key finding of this study is the improvement in lower limb muscle strength and physical function in patients after pullout repair for MMPRTs. A notable correlation was also identified between preoperative and postoperative lower limb muscle strength and physical function among patients with MMPRTs.

In many cases, it takes an amount of time from the onset of MMPRTs to surgery. Muscle strength was presumed to have decreased because of disuse during this period. Moreover, improved lower limb muscle strength has been reported to be associated with better meniscal function after MMPR repair [10]. In this study, the patients’ lower limb muscle strength significantly improved from the preoperative level to 1 year postoperatively. The patients regained the original level of meniscal function postoperatively. Moreover, continued physical therapy helped increase the activity, leading to enhanced muscle strength.

In this study, the TUG test was used to assess physical function. The TUG test is one of the diagnostic criteria for MADS. It is useful for a comprehensive assessment of physical function because it includes walking and activities of daily living, such as standing up, sitting up, and turning. Lower limb muscle strength is a major contributor to the quality of life and mobility in older individuals [12, 13]. Improvement in the strength of the quadriceps muscles is associated with an improved ability to rise from a chair [14]. In addition, knee extensor muscle strength has been correlated with the TUG time [15]. In this study, improvement in the lower limb muscle strength, especially knee extensor strength, may have influenced the postoperative improvement in TUG time.

The cutoff value for TUG in the diagnostic criteria for MADS was set at 11 s [16]. Others have reported a cutoff value of 9 s for TUG, which increases the risk of frailty and hospitalization [17]. In this study, the preoperative TUG was 12.3 s, meeting the diagnostic criteria for MADS. However, after surgical intervention and rehabilitation for MMPRTs, the TUG at 1 year postoperatively was 9.2 s, which did not meet the diagnostic criteria for MADS. Since a correlation has been shown between the TUG and leg muscle strength, continued efforts to improve the lower limb muscle strength are needed [18].

There have been several limitations in this study. Firstly, it was a retrospective study design. Secondly, the number of patients was relatively small, at 33. The actual power was over 80% in the post-hoc analysis of the correlation between postoperative knee extensor muscle strength and TUG. However, 13 patients were excluded, and the potential impact of selection bias should also be considered. Thirdly, the follow-up period was limited to only 1 year postoperatively. A longer-term evaluation is desirable for assessing physical function after meniscal repair. Fourthly, if specialized rehabilitation was continued or stopped beyond the initial period of 3 months after surgery, this may have affected the results of this study. Fifthly, the physical function assessment in this study was limited to the TUG test. Although the TUG test is a valuable assessment that evaluates multiple functions, including standing, sitting, walking, and changing directions, incorporating additional assessments of walking ability and motor function would have provided a more comprehensive evaluation. Sixthly, the use of muscle strength measurement devices like handheld dynamometers is limited in many clinical settings. However, by reporting a significant correlation between physical function and lower limb muscle strength, we consider that continuous lower limb muscle strengthening, regardless of the threshold or whether it is measured, can be expected to improve physical function.

## Conclusion

In patients with MMPRTs, surgery and rehabilitation significantly improved lower limb muscle strength and physical function. In addition, the TUG time improved from the preoperative value, which satisfied the MADS diagnostic criteria, to a value that did not satisfy the MADS diagnostic criteria at 1 year postoperatively. A notable correlation was observed between lower limb muscle strength and TUG, underscoring the importance of continued efforts to enhance the lower limb muscle strength through subsequent interventions.

## Data Availability

The datasets generated and analyzed during the current study are available from the corresponding author on reasonable request.
